# Knowledge and misconceptions about antibiotic use and antibiotic resistance among secondary school students in Riyadh, Saudi Arabia: implications for school- based antibiotic resistance education

**DOI:** 10.3389/fpubh.2026.1962239

**Published:** 2026-09-29

**Authors:** Sarah R. Alharbi, Lama Alzamil, Sara Alfaqeeh, Nouf Alhantoshi, Salem S. AlShammari, Meyad Alkarni, Sarah Almuhayya

**Affiliations:** Department of Clinical Laboratory Sciences, College of Applied Medical Sciences, King Saud University, Riyadh, Saudi Arabia

**Keywords:** AMR, antibiotic use, antimicrobial resistance, awareness, health education, secondary school students

## Abstract

**Background:**

Antibiotic resistance (AR) is a major global public health threat, and improving AR awareness among adolescents is a priority in efforts to combat it. However, adolescents remain an understudied population, particularly in Saudi Arabia. This study evaluated practices, knowledge, awareness, misconceptions, and attitudes regarding antibiotic use and AR among secondary school students in Riyadh, Saudi Arabia.

**Methods:**

A cross-sectional survey was conducted among 389 students from nine secondary schools (six public, two private, and one international) in Riyadh, between October 2024 and December 2024. A self-administered questionnaire assessed students' practices, knowledge, awareness, and attitudes regarding antibiotic use and AR. Associations with gender, school type, and participation in science or health clubs were examined using chi-square/Fisher's exact tests with false discovery rate adjustment and multivariable regression of composite knowledge scores.

**Results:**

Although most students reported positive attitudes toward responsible antibiotic use, substantial knowledge gaps were identified. Misconceptions were common regarding the treatment of viral infections, the biological basis of AR, and the role of antibiotic use in agriculture and food-producing animals. Correct responses to AR concept statements ranged from 8.7% to 42.2%. Students were more familiar with the terms antibiotic-resistant bacteria (49.4%), antibiotic resistance (44.5%), and drug resistance (40.6%) than with antimicrobial resistance (21.6%) or AMR (21.9%). Doctors were the most frequently reported source of information, whereas awareness campaigns were among the least frequently reported. After adjustment, club participation was not associated with the AR-concept score (β = 0.27, 95% CI −0.20 to 0.73), whereas female gender (β = 0.69, 95% CI 0.47 to 0.91) and private-school attendance (β = 0.30, 95% CI 0.06 to 0.54) were independently associated with higher knowledge of appropriate antibiotic use. Most students (61.7%) supported incorporating antibiotic use and AMR education into school curricula, primarily to increase awareness and knowledge and to protect individual and community health.

**Conclusions:**

Secondary school students in Riyadh demonstrated positive attitudes toward responsible antibiotic use but had important gaps in their understanding of AR. Strengthening school-based AR education, including appropriate antibiotic use, core AR concepts, and One Health concepts, could improve students' awareness and support national efforts to combat AR.

## 1 Introduction

Antibiotics are among the most important discoveries in modern medicine, saving millions of lives and transforming healthcare worldwide. They are essential for the safe delivery of numerous medical interventions, such as major surgery, organ transplantation, and cancer chemotherapy, where prevention and treatment of bacterial infections are critical (1). However, the effectiveness of antibiotics is increasingly threatened by antimicrobial resistance (AMR), a major global public health challenge. In 2019, bacterial AMR was directly responsible for an estimated 1.27 million deaths and contributed to an additional 4.95 million deaths worldwide (2). In addition to its impact on morbidity and mortality, AMR imposes a substantial economic burden on healthcare systems due to prolonged hospital stays, higher healthcare costs, and the use of more expensive antimicrobial therapies (3).

The misuse and overuse of antibiotics across the human, animal, and environmental sectors are among the main drivers of the emergence and spread of antibiotic resistance (AR). In human health, practices such as self-medication, unnecessary antibiotic use for viral infections, failure to complete prescribed courses, and sharing antibiotics contribute to the emergence and spread of resistant bacteria (4). Antibiotics are also widely used in food-producing animals and agriculture, contributing to the selection and dissemination of resistant bacteria within communities and the environment (5, 6).

Several studies and reports have shown that lack of knowledge and limited awareness of antibiotic use and AR are associated with inappropriate antibiotic use (7–9). Therefore, improving public understanding of antibiotics and AR is considered a key strategy for promoting responsible antibiotic use and slowing the emergence and spread of AR. In 2015, the World Health Organization (WHO) launched a global action plan on AMR (10). The first objective of this plan is to improve awareness and understanding of AMR through effective communication targeting various sectors of society, including human and animal health communities, students, and policymakers. The plan also recommends incorporating AMR into education and encourages countries to prioritize it on the political agenda.

In Saudi Arabia, the National Action Plan for Combating AMR was launched in 2017, followed by regulations prohibiting the sale of antibiotics without a prescription in 2018 (11). Before implementing this restriction policy, self-medication and non-prescription access to antibiotics were common practices among the public (12, 13). In alignment with the WHO objectives, one of the main objectives of the Saudi national action plan is to increase national awareness of this public health problem. To achieve this objective, the Saudi national plan advocates assessing public awareness about AMR and conducting surveys on public awareness of the use and misuse of antibiotics (14).

Several national studies have evaluated knowledge and awareness of antibiotic use and AR among adults, including the general public, healthcare professionals, and university students, across different regions of Saudi Arabia. These studies have reported varying levels of knowledge and have mostly identified important misconceptions and knowledge gaps regarding antibiotic use and resistance (15–21). However, little attention has been given to adolescents, a critical population for AR education (22).

Attitudes and health behaviors established during adolescence often persist into adulthood (23). Schools therefore provide an ideal setting for promoting long-term improvements in health knowledge and behaviors. Secondary school students, as future healthcare consumers and potential healthcare professionals, are a key target for AR education. Enhancing their knowledge of appropriate antibiotic use and AR early on can promote responsible antibiotic use that lasts into adulthood. Moreover, students often share what they learn with their families and communities, extending the potential public health benefits of school-based education beyond just the individual (23). Therefore, assessing secondary school students' knowledge is essential for identifying misconceptions and curriculum gaps, which can guide the development of future educational strategies.

Evidence regarding AR knowledge among school-aged populations remains limited internationally and particularly in Saudi Arabia (22, 24–27). To our knowledge, only one previous study has evaluated AMR awareness among high school students in Saudi Arabia. Almutairi et al. (27) conducted a quasi-experimental study among 240 students from three high schools in Riyadh to evaluate the effect of a 15-min educational lecture on knowledge and perceptions of AMR. The study assessed antibiotic-use practices and several aspects of knowledge and misconceptions regarding antibiotic use and resistance and demonstrated improvements following the intervention. However, its primary focus was evaluating intervention effectiveness rather than providing a comprehensive characterization of students' baseline knowledge and awareness. The present study extends the limited Saudi evidence by assessing a broader range of domains, including knowledge of conditions treatable with antibiotics, familiarity with multiple AR-related terms and sources of information, awareness of antibiotic use in agriculture and food-producing animals, and perceptions of school-based antibiotic education. It also includes students from public, private, and international schools and examines differences according to school type and participation in science or health clubs. Thus, the present study provides a broader assessment of students' knowledge, misconceptions, and awareness across these domains and educational settings.

This study aimed to evaluate secondary school students' knowledge, awareness, misconceptions, and attitudes toward antibiotic use and AR in Riyadh, Saudi Arabia. It also aimed to identify knowledge gaps to guide future educational efforts and to incorporate AR education into school curricula.

## 2 Materials and Methods

### 2.1 Study design and setting

We conducted a cross-sectional survey targeting secondary school students enrolled in Grades 10–12 in Riyadh, Saudi Arabia. Schools were recruited by convenience; to broaden coverage, invitations were sent to schools in different geographical areas of Riyadh and to each of the three school types operating in the city (public, private, and international).

Participation at the school level was voluntary, and schools were included based on their agreement to participate. The inclusion of an international school allowed students following a curriculum other than that of the Saudi Ministry of Education to be included, although this group was small. A total of nine secondary schools participated, comprising six public, two private, and one international school. All students in the eligible grades at participating schools were invited to complete the questionnaire. A total of 389 students completed the questionnaire and were included in the final analysis.

### 2.2 Sample size

The minimum sample size was estimated using the formula for a single proportion, *n* = Z^2^*p*(1 – p)/e^2^. Because the prevalence of adequate AR knowledge among Saudi secondary school students was unknown, p was set at 0.5 to yield the most conservative estimate, with a 95% confidence level (Z = 1.96) and a 5% margin of error, giving a minimum of 385 participants. Because the target population (secondary school students in Riyadh) is large, no finite-population correction was applied. The final sample of 389 exceeded this minimum. The calculation did not account for clustering of students within schools, which is acknowledged as a limitation.

### 2.3 Questionnaire

The questionnaire (**Supplementary file, Questionnaire S1**) was adapted from the World Health Organization (WHO) Multi-country Public Awareness Survey on Antibiotic Resistance (7) and from the questionnaire developed by Ambusaidi et al. for secondary school students in Oman, which was based on the WHO instrument (24). Minor modifications were made to reflect the Saudi context, including the addition of demographic questions (e.g., nationality, school type, grade level, and participation in school health or science clubs), adaptation of country-specific references to Saudi Arabia, and brief explanatory definitions for selected diseases included (Question 9).

The questionnaire was translated from English into Arabic by the first author (S.R.A). The English and Arabic versions of the questions were presented side by side in a bilingual questionnaire. Both language versions were reviewed by a professional linguist to ensure linguistic accuracy, clarity, and conceptual consistency. The questionnaire was subsequently pilot-tested with 10 secondary school students to assess readability, comprehension, and the clarity of both language versions. Based on participant feedback, minor wording revisions were made to improve clarity, and brief explanatory definitions were added for selected disease terms that students found unfamiliar before the main study was administered.

### 2.4 Data collection

Data were collected over a three-month period, from October 2024 to December 2024. During scheduled visits to the participating schools, members of the research team administered the questionnaire. Students with access to electronic devices received a Microsoft Forms survey link to complete the questionnaire electronically, whereas students without access to an electronic device received an identical paper-based questionnaire. Before administering the questionnaire, students received a brief explanation of the study objectives and instructions on how to complete it. Participants completed the questionnaire independently without discussion, while members of the research team were present only to distribute the questionnaires, answer procedural questions, and collect the completed paper questionnaires. Paper responses were manually entered into the electronic dataset and checked against the original forms by a second team member before being merged with the Microsoft Forms responses.

### 2.5 Ethical considerations

The study received ethical approval from the Institutional Review Board of King Saud University (Ref. No. KSU-HE-24-423) and permission from the Ministry of Education (Ref. No. 4501166486) and the participating schools.

Participation was voluntary. Written informed consent was obtained from participants' parents or legal guardians in accordance with the Ministry of Education's requirements. In addition, all students received a bilingual (Arabic/English) information statement at the start of the questionnaire describing the study purpose, the voluntary nature of participation, the right to withdraw at any time without consequence, and the anonymity of responses. Students aged 18 years or older indicated their consent through their voluntary decision to complete the questionnaire. Questionnaire responses were anonymous, and all data were securely stored, with access restricted to the research team.

### 2.6 Statistical analysis

#### 2.6.1 Data handling and scoring

Categorical variables were summarized as frequencies and percentages. Knowledge items were scored dichotomously as correct or incorrect against the predefined correct response for each statement, with “I don't know” responses scored as incorrect, and the proportion of correct responses was calculated for the total sample and for each subgroup. For the multiple-response item on conditions treatable with antibiotics, participants could select more than one option; percentages were therefore calculated using the total number of participants (*N* = 389) as the denominator and do not sum to 100%. For conditionally administered items on the most recent course of antibiotics, percentages were calculated using valid responses only (*n* = 222–223), and the number of non-respondents is reported alongside each item. Items with missing responses were analyzed using available data; no imputation was performed. Attitude items were reported as the distribution of responses across the five-point Likert scale.

Two composite knowledge scores were constructed: an AR-concept score (0–10), calculated as the number of correct responses to the ten AR-concept statements (Table 1), and an appropriate-use score (0–3), calculated as the number of correct responses to the three appropriate-use items (Table 2). Internal consistency was assessed with the Kuder–Richardson 20 coefficient and was acceptable for exploratory composite measures (KR-20 = 0.62 for the AR-concept score and 0.64 for the appropriate-use score), consistent with the items assessing related but distinct concepts.

**Table 1:** Knowledge of key antibiotic resistance concepts among secondary school students (*n* = 389).

Statement	Correct response	*n* (%) correct
Antibiotic resistance occurs when your body becomes resistant to antibiotics, and they no longer work as well	Wrong	34 (8.7)
A lot of types of infections have become increasingly resistant to antibiotics	Correct	149 (38.3)
If bacteria are resistant to antibiotics, it can be very difficult or impossible to treat the infections they cause	Correct	164 (42.2)
Antibiotic resistance is an issue that could affect me or my family	Correct	154 (39.6)
Antibiotic resistance is an issue in other countries but not in Saudi Arabia	Wrong	105 (27.0)
Antibiotic resistance is only a problem for people who take antibiotics regularly	Wrong	59 (15.2)
Bacteria that are resistant to antibiotics can be spread from person to person	Correct	132 (33.9)
Antibiotic-resistant infections could make medical procedures such as surgery, organ transplantation, and cancer treatment much more dangerous	Correct	157 (40.4)
Antibiotic resistance can be overcome by increasing the dose of the medication	Wrong	135 (34.7)
Viral infections can be cured using antibiotics	Wrong	74 (19.0)

Statements marked “Wrong” are misconceptions; the correct response is to reject them (select “Wrong”). Statements marked “Correct” are true; the correct response is to select “Correct.” Percentages represent correct responses in either direction. “I don't know” responses were scored as incorrect.

**Table 2:** Correct responses to questions assessing appropriate antibiotic use according to participant characteristics.

Item	Participant characteristic	Correct responses, *n*/*N* (%)	*p*-value	*q*-value
	Overall	183/389 (47.0)	—	—
When do you think you should stop taking antibiotics once you've begun treatment? Correct response: according to instructions	**Gender**		< 0.001	0.008
	Female	108/191 (56.5)		
	Male	75/198 (37.9)		
	**School type**		0.300	0.641
	Public	120/270 (44.4)		
	Private	57/108 (52.8)		
	International	6/11 (54.5)		
	**Science/health club participation**		0.466	0.783
	Yes	108/238 (45.4)		
	No	75/151 (49.7)		
	Overall	227/389 (58.4)	—	
It's okay to use antibiotics that were given to a friend or family member, as long as they were used to treat the same illness. Correct response: FALSE	**Gender**		< 0.001	0.008
	Female	143/191 (74.9)		
	Male	84/198 (42.4)		
	**School type**		0.072	0.346
	Public	148/270 (54.8)		
	Private	73/108 (67.6)		
	International	6/11 (54.5)		
	**Science/health club participation**		0.598	0.819
	Yes	136/238 (57.1)		
	No	91/151 (60.3)		
	Overall	120/389 (30.8)	—	
It's okay to buy the same antibiotics, or request these from a doctor, if you're sick and they helped you get better when you had the same symptoms before Correct response: FALSE	**Gender**		< 0.001	0.008
	Female	76/191 (39.8)		
	Male	44/198 (22.2)		
	**School type**		0.141	0.373
	Public	75/270 (27.8)		
	Private	41/108 (38.0)		
	International	4/11 (36.4)		
	**Science/health club participation**		0.652	0.819
	Yes	71/238 (29.8)		
	No	49/151 (32.5)		

*n* = number of correct responses; *N* = total number of participants in each subgroup. The direction of the correct response differs by item: for the first item the correct answer is “according to the instructions”; the second and third items are false statements, so the correct response is “False.” Percentages therefore represent the proportion of students giving the correct answer, not the proportion agreeing with the statement. *p*-values are from Fisher's exact test (gender and club participation) or Pearson's chi-square test (school type); *q*-values are Benjamini–Hochberg adjusted *p*-values across all 47 subgroup comparisons (Table 2 and Supplementary Tables S1 and S2). The international-school subgroup comprised 11 students; its estimates are imprecise and school-type *p*-values should be interpreted with caution.

#### 2.6.2 Statistical tests

Associations between participant characteristics and dichotomous outcomes were assessed using Fisher's exact test for two-group comparisons (gender and club participation) and Pearson's chi-square test for the three-category school-type comparisons. Correct responses to the appropriate-use items and awareness of each AMR-related term were compared by gender, school type, and participation in science or health clubs (Table 2 and Supplementary Tables S1); correct responses to the 10 AR-concept statements were compared by school type and club participation (Supplementary Table S2), with gender examined at the composite-score level in the multivariable model described below. Because the international-school subgroup was small, school-type comparisons had limited power to detect differences involving this group, and estimates for this subgroup are imprecise. Item-level comparisons were considered exploratory; *p*-values were adjusted for multiple comparisons using the Benjamini–Hochberg false discovery rate procedure across all 47 subgroup tests (Table 2, Supplementary Tables S1, and S2), and an adjusted q-value of less than 0.05 was considered statistically significant.

To disentangle the effects of gender, school type, and club participation, which may be correlated, each composite score was modeled as the dependent variable in a multivariable linear regression with gender, school type (private vs. public and international vs. public, with the international school retained as a separate indicator), and club participation entered simultaneously, using heteroscedasticity-robust (HC3) standard errors. Results are presented as adjusted regression coefficients (β) with 95% confidence intervals; a Poisson model for the AR-concept score and a sensitivity analysis excluding the international school gave the same conclusions. Students were nested within nine schools; analyses did not adjust for clustering because of the small number of clusters, and standard errors may therefore be slightly underestimated.

All tests were two-tailed, and a *p*-value of less than 0.05 was considered statistically significant for the pre-specified multivariable analyses. Responses to the open-ended item on the perceived importance of learning about antibiotic use in schools were analyzed using qualitative content analysis. Responses were read repeatedly for familiarization, coded inductively, and grouped into themes; coding was performed by MA and reviewed by LA, and disagreements were resolved by discussion. Responses that were unclear, did not provide a meaningful reason, or indicated that the participant did not know were categorized separately.

Data were analyzed using GraphPad Prism (version 9.3; GraphPad Software, LLC, San Diego, CA, USA) and Microsoft Excel (Microsoft Corporation, Redmond, WA, USA), and Python 3 (statsmodels version 0.14) for the false discovery rate adjustment and multivariable models.

## 3 Results

### 3.1 Sociodemographic characteristics of the participants

A total of 389 secondary school students completed the questionnaire (Table 3). The study population comprised 198 (50.9%) males and 191 (49.1%) females. Participants ranged in age from 14 to 20 years, with most being 16 years old (36.5%), followed by 17 years (31.1%) and 15 years (23.7%). The majority of participants were Saudi nationals (89.7%).

**Table 3:** Demographic characteristics of participants (*n* = 389).

Sociodemographic characteristics	*n* (%)
Gender distribution	
Male	198 (50.9%)
Female	191 (49.1%)
Total	389
Age distribution	
15	92 (23.7%)
16	142(36.5%)
17	121 (31.1%)
18	23(5.9%)
14	4 (1%)
19	6 (1.5%)
20	1 (0.2%)
Nationality	
Saudi	349 (89.7%)
Non-Saudi	40 (10.3%)
School grade	
10	155(39.8%)
12	128 (32.9%)
11	106 (27.2%)
School type	
Public	270 (69.4%)
Private	108 (27.8%)
International	11 (2.8%)
Participation in science/health clubs	
Yes	238 (61.2%)
No	151 (38.8%)

Students were enrolled in all three secondary school grades, with 39.8% in Grade 10, 27.2% in Grade 11, and 32.9% in Grade 12. Most participants attended public schools (69.4%), while 27.8% attended private schools and 2.8% attended international schools. Overall, 61.2% reported participating in school science or health clubs. Club participation was more common among private-school students (75.9%) than among public-school (56.3%) or international-school (36.4%) students.

### 3.2 Antibiotic use practices

Table 4 summarizes participants' previous antibiotic use and related practices. Nearly one-quarter of participants (23.9%) reported taking antibiotics in the previous month, while 17.0% had used antibiotics in the previous 6 months. In contrast, 39.6% were unable to recall when they had last taken antibiotics, whereas only 3.1% reported never having taken antibiotics.

**Table 4:** Antibiotic use practices among secondary school students.

Item	Response	*n* (%)
Last took antibiotics (*n* = 389)	Past month	93 (23.9)
	Past 6 months	66 (17.0)
	Past year	33 (8.5)
	More than a year ago	31 (8.0)
	Never consumed antibiotics	12 (3.1)
	Don't remember	154 (39.6)
Obtained antibiotics through a doctor/medical prescription (*n* = 222)	Yes	194 (87.4)
	No	19 (8.6)
	Don't know/don't remember	9 (4.1)
Received advice from a doctor, nurse, or pharmacist on how to take antibiotics (*n* = 223)	Yes	180 (80.7)
	No	21 (9.4)
	Don't remember	22 (9.9)
Received instructions from parents on how to take antibiotics (*n* = 222)	Yes	178 (80.2)
	No	28 (12.6)
	Don't remember	16 (7.2)
Source of antibiotics (*n* = 223)	Medical store or pharmacy	179 (80.3)
	Saved up from a previous time	13 (5.8)
	Friend or family member	2 (0.9)
	The internet	2 (0.9)
	Somewhere/someone else	3 (1.3)
	Don't remember	24 (10.8)

Follow-up items were completed only by participants who reported previous antibiotic use that they could recall. Students who had never taken antibiotics (*n* = 12) or could not recall their most recent antibiotic use (*n* = 154) were not eligible to answer these items. Percentages were calculated using valid responses (*n* = 222–223), with the difference due to one missing response.

Among participants with a history of antibiotic use and who completed the follow-up questions, the majority (87.4%) received antibiotics via a doctor's prescription. Most also reported receiving advice from a doctor, nurse, or pharmacist on how to take the antibiotics (80.7%) and receiving instructions from their parents (80.2%). Antibiotics were obtained predominantly from a medical store or pharmacy (80.3%), whereas relatively few participants reported using antibiotics saved from a previous illness (5.8%), obtained from a friend or family member (0.9%), the internet (0.9%), or another source (1.3%).

### 3.3 Knowledge of appropriate antibiotic use

Knowledge of appropriate antibiotic use varied, with correct response rates ranging from 30.8% to 58.4% (Table 2). Less than half of participants (47.0%) correctly identified that antibiotics should be taken according to the prescribed course, and only 30.8% correctly recognized that antibiotics should not be requested or reused for similar symptoms after a previous treatment. In contrast, 58.4% correctly recognized that antibiotics should not be shared with friends or family members, even when treating the same illness. Female students demonstrated significantly higher levels of knowledge about appropriate antibiotic use than males across all three questions (all *p* < 0.001; all *q* = 0.008). No significant differences were observed by school type (noting that the international-school estimates are based on only 11 students) or by participation in science or health clubs. The mean appropriate-use score was 1.36 (SD 1.11; median 1) out of 3. In the multivariable model (Table 5), female gender remained independently associated with a higher score (β = 0.69, 95% CI 0.47 to 0.91, *p* < 0.001), and attending a private school was also independently associated with a higher score (β = 0.30, 95% CI 0.06 to 0.54, *p* = 0.013), whereas club participation was not (β = −0.01, 95% CI −0.24 to 0.22, *p* = 0.95).

**Table 5:** Multivariable regression of composite knowledge scores on participant characteristics.

Characteristic	AR-concept score (0–10): adjusted estimate (95% CI)	*p*	Appropriate-use score (0–3): adjusted estimate (95% CI)	*p*
Gender (female vs. male)	−0.08 (−0.51 to 0.34)	0.70	0.69 (0.47 to 0.91)	< 0.001
School type (private vs. public)	0.25 (−0.25 to 0.76)	0.33	0.30 (0.06 to 0.54)	0.013
Science/health club (yes vs. no)	0.27 (−0.20 to 0.73)	0.26	−0.01 (−0.24 to 0.22)	0.95

Values are adjusted regression coefficients (β) with 95% confidence intervals from multivariable linear regression with heteroscedasticity-robust (HC3) standard errors, with all characteristics entered simultaneously. The international school (*n* = 11) was included as a separate indicator (international vs. public: AR-concept score β = 0.28, 95% CI −1.10 to 1.66, *p* = 0.69; appropriate-use score β = −0.12, 95% CI −0.90 to 0.66, *p* = 0.77). Model *R*^2^ = 0.009 (F-test *p* = 0.52) for the AR-concept score and 0.112 (F-test *p* < 0.001) for the appropriate-use score.

### 3.4 Knowledge of medical conditions that are treatable with antibiotics.

Students were asked to select, from a list of 14 conditions, all those they believed could be treated with antibiotics. The most frequently selected conditions were cold and flu (69.9%), fever (67.9%), and throat infection (60.4%), followed by headache (42.9%) and body aches (37.8%) (Figure 1). Cold and flu are viral illnesses, so antibiotics are not suitable treatment, whereas fever, headache, and body aches are symptoms rather than specific infections.

**Figure 1:**
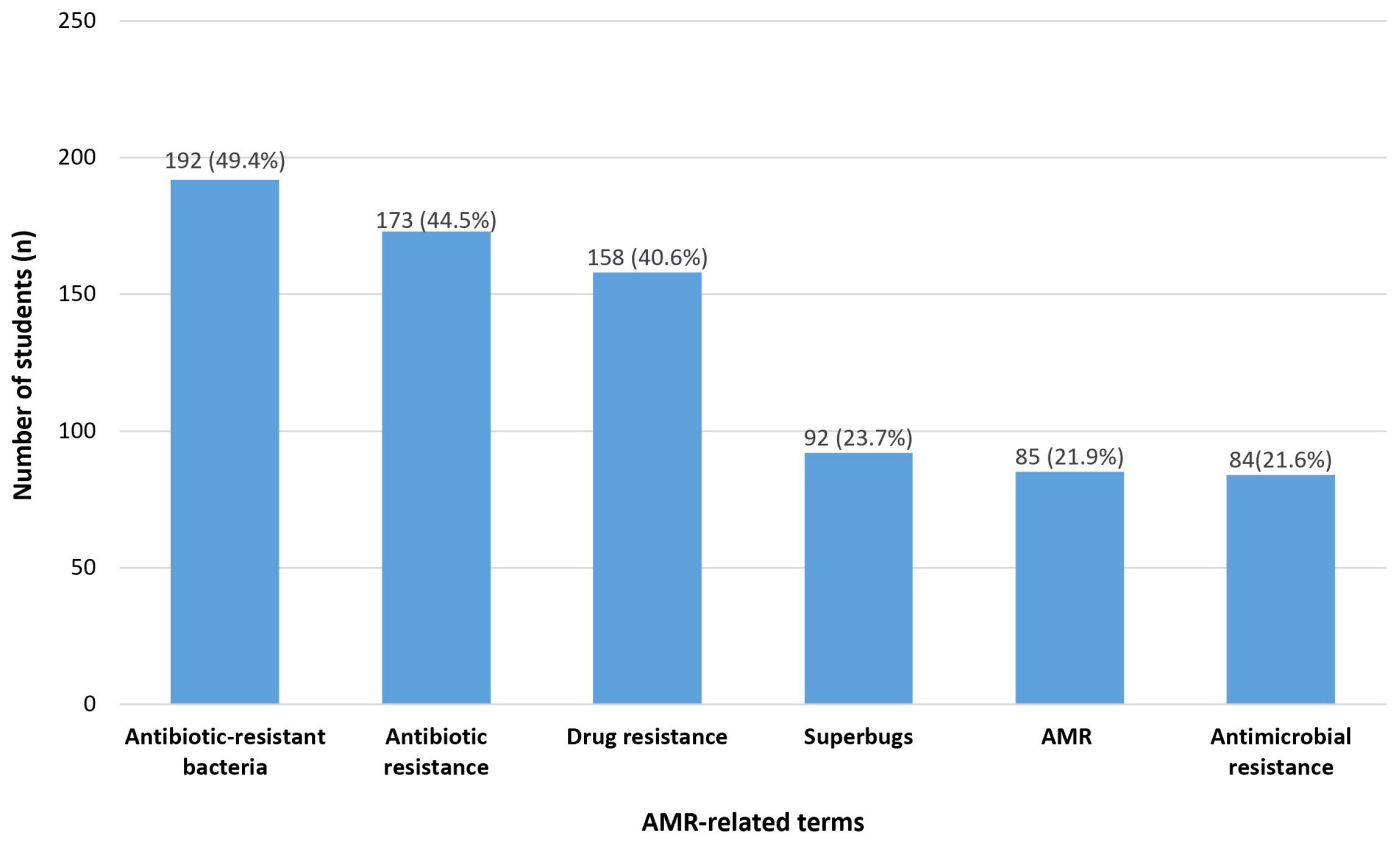
Diseases that secondary school students believed could be treated with antibiotics (*N* = 389).

Recognition of bacterial infections that commonly require antibiotic treatment was generally low. Only 28.8% of students recognized skin or wound infections as treatable with antibiotics, followed by urinary tract infections (28.5%), brucellosis (27.2%), gonorrhea (22.9%), and tuberculosis (18.5%).

Responses for throat infection and diarrhea were classified as partially correct, as antibiotics are appropriate only for specific bacterial causes of these conditions.

Additionally, 20.8%, 20.3%, and 17.5% of participants identified acquired immunodeficiency syndrome (AIDS), malaria, and measles, respectively, as conditions that can be treated with antibiotics. AIDS and measles are viral diseases, whereas malaria is caused by parasites.

### 3.5 Awareness of antibiotic use in the agricultural sector

Students were asked whether they believed antibiotics are used on a large scale in the agricultural sector in Saudi Arabia, including in food-producing animals (Table 6). Half of the participants (50.1%) reported that they did not know whether antibiotics are used in agriculture, while 38.8% believed they are used on a large scale. Only 11.1% believed that antibiotics are not used in the agricultural sector.

**Table 6:** Students' awareness of antibiotic use in the agricultural sector in Saudi Arabia (*N* = 389).

Response	*n* (%)
Yes	151 (38.8)
No	43 (11.1)
Don't know	195 (50.1)
Total	389 (100.0)

### 3.6 Awareness of antibiotic resistance-related terms

Students' awareness of six antibiotic resistance (AR) -related terms is presented in Figure 2. The term “antibiotic-resistant bacteria” was the most frequently recognized, with 192 (49.4%) students reporting having previously heard of it. This was followed by “antibiotic resistance” (173, 44.5%) and “drug resistance” (158, 40.6%). In contrast, awareness was considerably lower for the terms “superbugs” (92, 23.7%), “AMR” (85, 21.9%), and “antimicrobial resistance” (84, 21.6%).

**Figure 2:**
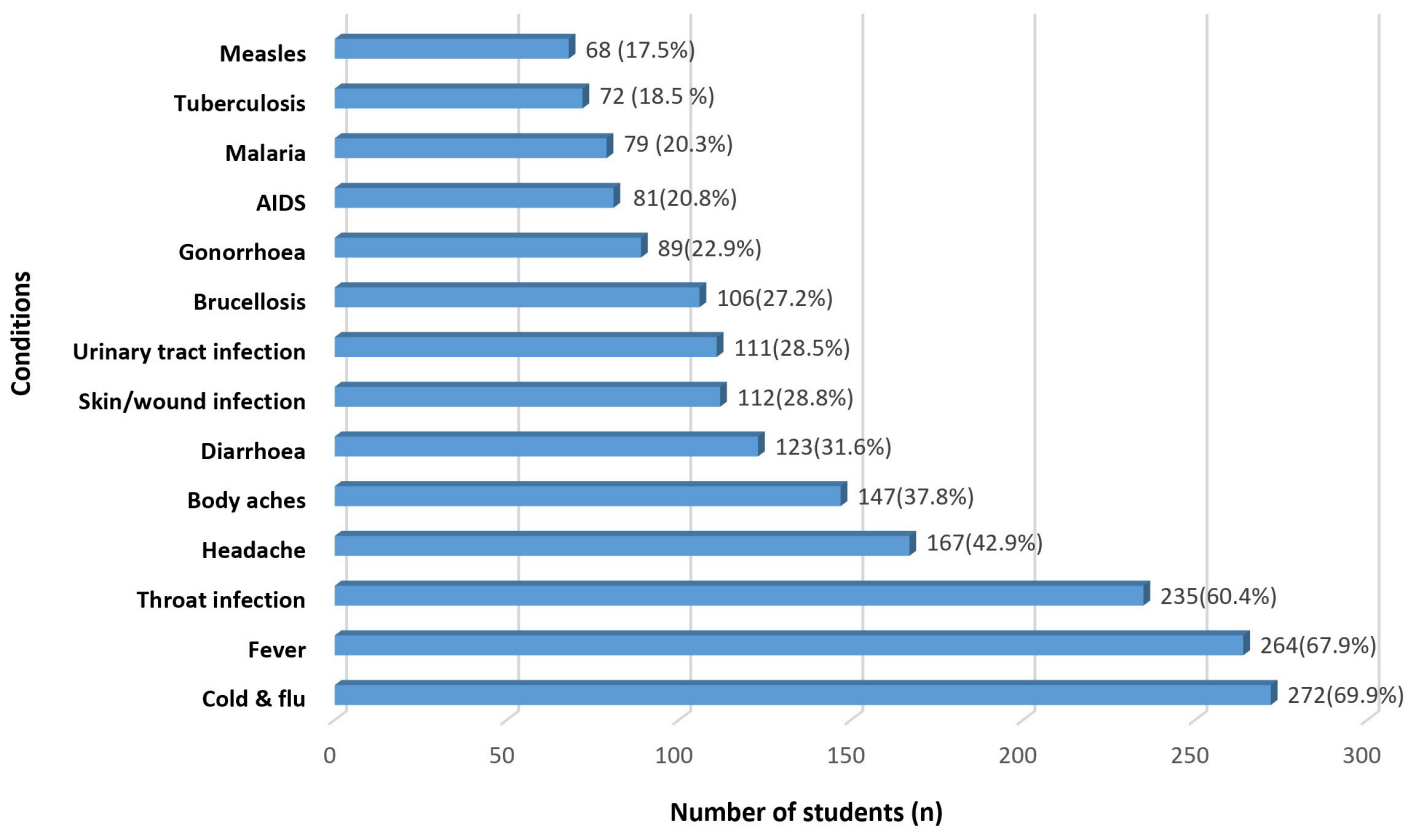
Students' awareness of antibiotic resistance-related terms (*N* = 389).

Among students who were aware of each AR-related term, doctors were the most commonly reported source of information for antibiotic resistance, antimicrobial resistance, and AMR, whereas school curriculum was the leading source of information for antibiotic-resistant bacteria (Table 7; full breakdown by source in Supplementary Figure S1). Media (television, radio, newspapers, or other media, including social media) was the most frequently reported source for drug resistance, and was also commonly reported for the remaining AR-related terms. Overall, friends or relatives and awareness campaigns were among the least frequently reported sources of information across all AR-related terms.

**Table 7:** Two most frequently reported sources of information for each antibiotic resistance-related term, among students aware of the term.

Term	Students aware, *n*	Most frequent source, *n* (%)	Second source, *n* (%)
Antibiotic-resistant bacteria	192	School curriculum, 76 (39.6)	Media, 65 (33.9)
Antibiotic resistance	173	Doctor, 82 (47.4)	Media, 63 (36.4)
Drug resistance	158	Media, 65 (41.1)	Doctor, 54 (34.2)
Superbugs	92	Media, 31 (33.7)	School curriculum, 30 (32.6); Doctor, 30 (32.6)
AMR	85	Doctor, 33 (38.8)	Media, 26 (30.6)
Antimicrobial resistance	84	Doctor, 41 (48.8)	Media, 32 (38.1)

Percentages use the number of students aware of each term as the denominator; participants could select more than one source. Media includes television, radio, newspapers, and social media. The full breakdown across all eight sources is provided in Supplementary Figure S1.

In exploratory subgroup analyses, awareness of the term “antibiotic resistance” was higher among students who participated in science or health clubs than among those who did not (50.4% vs. 35.1%, *p* = 0.003; *q* = 0.035) (Supplementary Table 1 S1). Similarly, awareness of drug resistance was higher among students participating in science or health clubs (45.4% vs. 33.1%, *p* = 0.020), but this difference did not remain significant after adjustment for multiple comparisons (*q* = 0.188). Awareness of the remaining AR-related terms, including superbugs, antimicrobial resistance, AMR, and antibiotic-resistant bacteria, did not differ by club participation, and no term differed by gender or school type (all q > 0.05). School-type comparisons should be interpreted with caution given the small international-school subgroup.

### 3.7 Understanding the issue of antibiotic resistance

Students' understanding of key AR concepts was assessed using a series of statements with three response options (correct, wrong, or I don't know). The proportion of students selecting the correct response for each statement is presented in Table 1. Correct response rates ranged from 8.7% to 42.2%. The mean AR-concept score was 2.99 (SD 2.11; median 3, IQR 1–5; range 0–10) out of 10, and 61 students (15.7%) answered none of the ten statements correctly.

The highest level of correct knowledge concerned the consequences of AR and how widely it can affect people. The statement most often answered correctly was that infections caused by antibiotic-resistant bacteria can be difficult or impossible to treat (42.2%), followed by the recognition that antibiotic-resistant infections could make medical procedures such as surgery, organ transplantation, and cancer treatment more dangerous (40.4%), and that antibiotic resistance could affect themselves or their families (39.6%). Approximately 38.3% of students correctly recognized that many infections have become increasingly resistant to antibiotics, while 37.5% correctly identified that antibiotic resistance is not limited to people who take antibiotics regularly.

Knowledge regarding the mechanisms and transmissibility of antibiotic resistance was generally limited. Only 33.9% correctly recognized that antibiotic-resistant bacteria can spread from person to person, 34.7% correctly rejected the misconception that AR can be overcome by increasing the antibiotic dose, and 27.0% correctly recognized that AR is also a problem in Saudi Arabia. The lowest level of knowledge was observed for the misconception that the human body, rather than bacteria, becomes resistant to antibiotics, with only 8.7% of students answering this statement correctly. In addition, only 19.0% correctly recognized that antibiotics cannot be used to treat viral diseases.

In exploratory item-level analyses (Supplementary Table S2), participation in science or health clubs was associated with higher correct response rates for two statements: recognition that many infections have become increasingly resistant to antibiotics (42.9% vs. 31.1%, *p* = 0.025), and awareness that antibiotic-resistant bacteria can spread from person to person (38.2% vs. 27.2%, *p* = 0.028). Neither remained significant after adjustment for multiple comparisons (both *q* = 0.188). Rejection of the misconception that antibiotic resistance affects only regular antibiotic users did not differ by club participation (15.1% vs. 15.2%, *p* = 1.00). No differences according by school type were observed for any of the statement, although the international-school group was too small for these comparisons to be informative.

In the multivariable model of the AR-concept score (Table 5), club participation was not independently associated with a higher score after adjustment for gender and school type (β = 0.27, 95% CI −0.20 to 0.73, *p* = 0.26). Gender (β for female = −0.08, 95% CI −0.51 to 0.34, *p* = 0.70) and school type (private vs. public: β = 0.25, 95% CI −0.25 to 0.76, *p* = 0.33) were also not associated with the score, and the model explained less than 1% of its variance (*R*^2^ = 0.009).

### 3.8 Attitudes toward how to address the issue of antibiotic resistance

Overall, students demonstrated positive attitudes toward appropriate antibiotic use (Figure 3) and (Supplementary Table S3). Combined agreement (agree strongly or agree slightly) exceeded 59% for all eight statements, and strong agreement was the most frequent response for every item.

**Figure 3:**
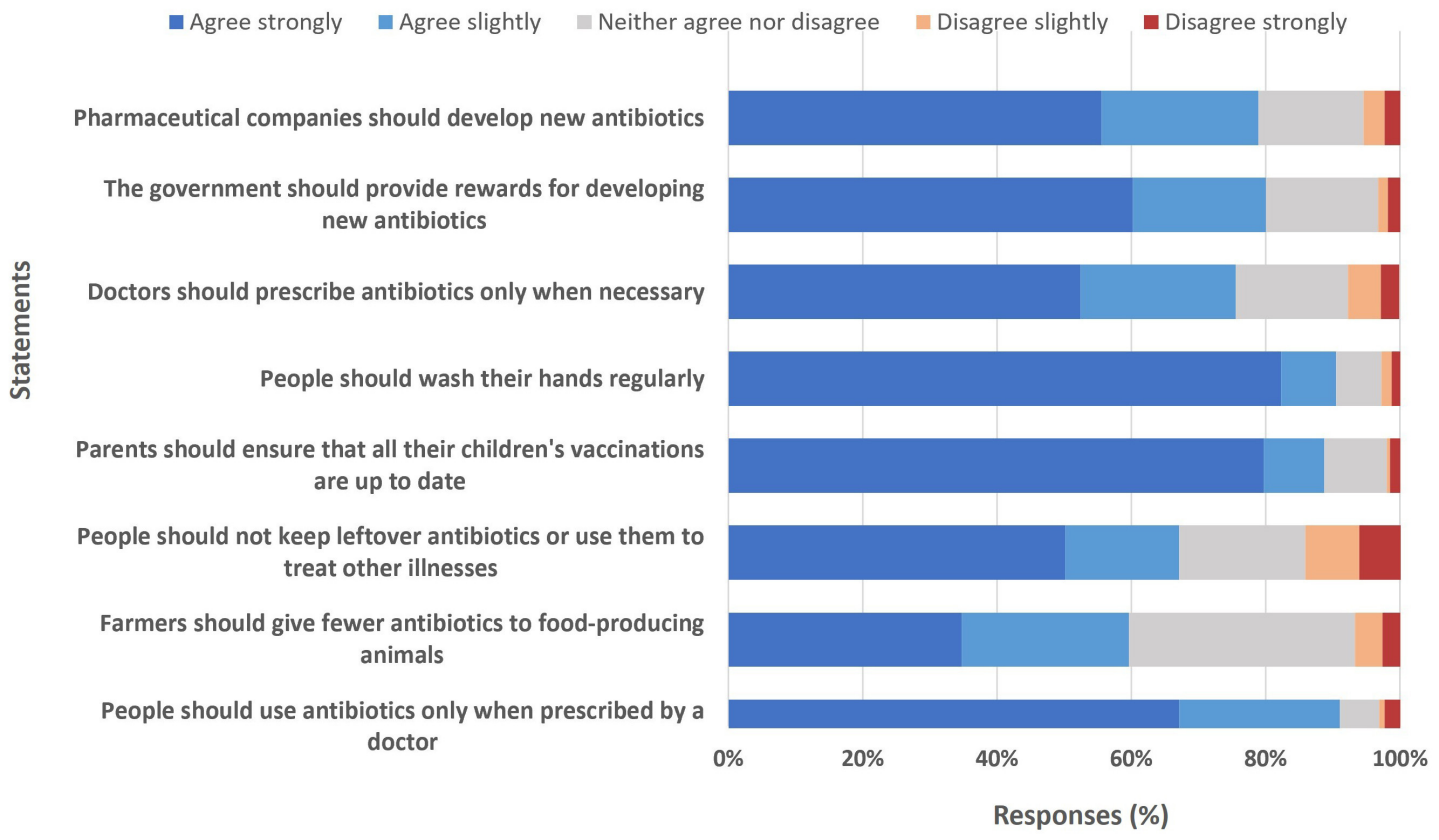
Students' attitudes toward how to address the issue of antibiotic resistance (*N* = 389).

Strong agreement was highest for personal preventive and prescribing behaviors. Most students strongly agreed that people should wash their hands regularly (82.3%), that parents should ensure their children's vaccinations are up to date (79.7%), and that antibiotics should be used only when prescribed by a doctor (67.1%). Strong agreement was also substantial for statements on responsible prescribing and antibiotic development: that governments should provide rewards for developing new antibiotics (60.2%), that pharmaceutical companies should develop new antibiotics (55.5%), and that doctors should prescribe antibiotics only when necessary (52.4%). For these items, neutral responses were relatively low (5.9–16.7%) and strong disagreement rarely exceeded 3%.

The two remaining statements received mixed reactions. Half of the students strongly agreed that leftover antibiotics should not be kept or reused (50.1%), but this statement also had the highest levels of disagreement (8.0% slightly and 6.2% strongly). The statement about farmers administering fewer antibiotics to food-producing animals had the lowest strong agreement at 34.7%, and it also had the largest neutral response, with 33.7% of students neither agreeing nor disagreeing.

### 3.9 Attitudes toward the seriousness and personal relevance of antibiotic resistance

Students' attitudes toward the seriousness of AR and its relevance to them are presented in Figure 4 and (Supplementary Table S4). Responses varied across the statements, with relatively high proportions of neutral responses for several items. Strong agreement was highest for the shared responsibility and concern statements. Most students strongly agreed that everyone should take responsibility for using antibiotics responsibly (60.4%), while 42.9% strongly agreed that they were worried about the impact of antibiotic resistance on their own and their family's health. In addition, 40.6% of students strongly agreed that medical experts will find a solution to antibiotic resistance before it becomes too serious.

**Figure 4:**
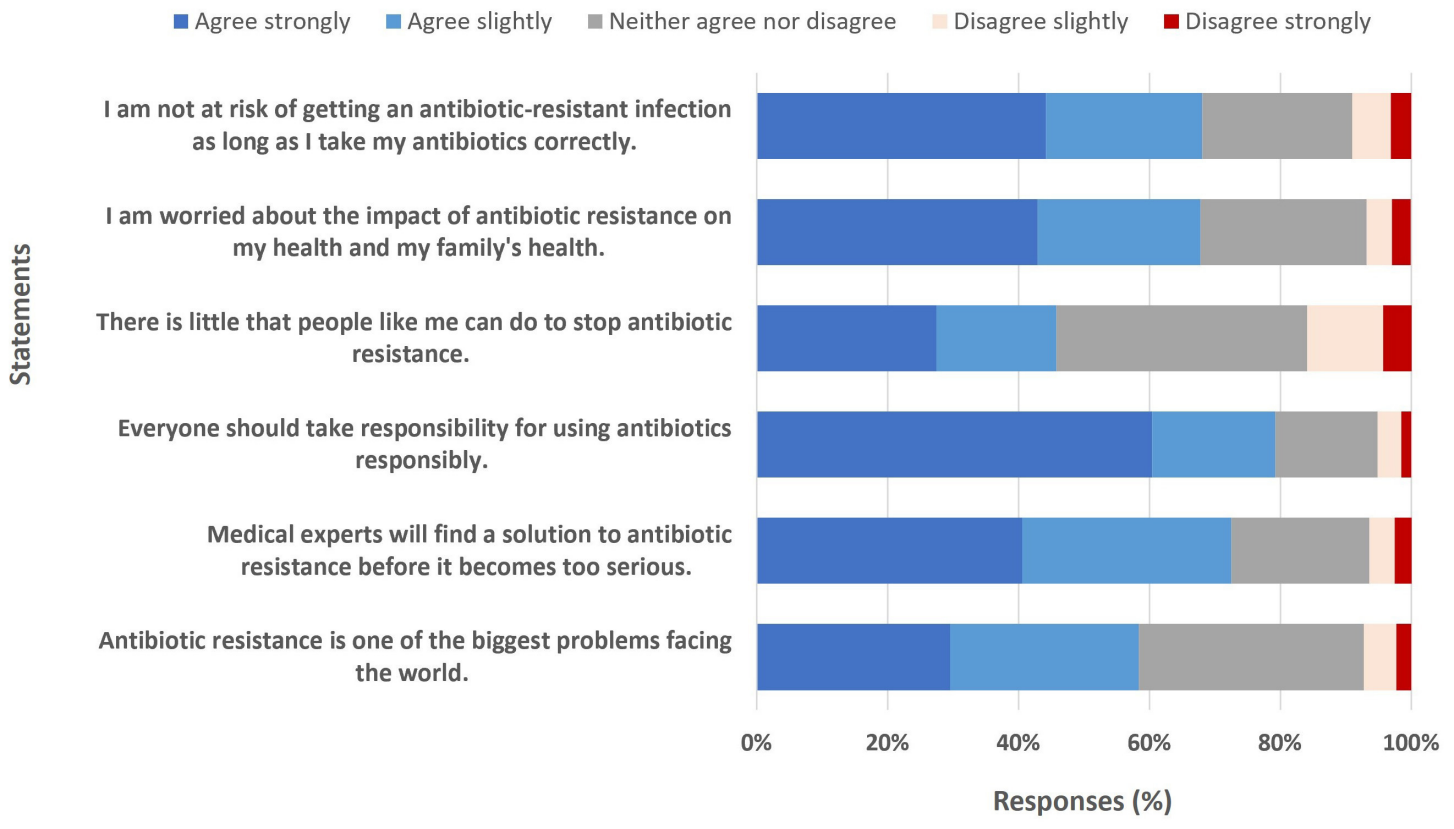
Students' attitudes toward the seriousness and personal relevance of antibiotic resistance (*N* = 389).

Perceptions of the global importance of AR were more uncertain. Only 29.6% of students strongly agreed that AR is one of the world's greatest health problems, while 34.4% neither agreed nor disagreed.

Responses to the statements addressing personal risk and self-efficacy were similarly mixed. Almost half of the students strongly agreed that they were not at risk of an antibiotic-resistant infection provided they took their antibiotics correctly (44.2%), and overall agreement reached 68.1% when those who agreed slightly were included. For the statement that there is little that people like them can do to stop AR, 27.5% strongly agreed, and 38.3% neither agreed nor disagreed, which was the highest proportion of neutral responses across all statements in this section.

### 3.10 Perceptions on AMR education

Students were asked whether they thought it was important to learn about the use of antibiotics in schools. Most students (61.7%) thought it was important, whereas 12.6% did not consider it important and 25.7% selected “I do not know” (Table 8).

**Table 8:** Students' perceptions of the importance of learning about antibiotic use in schools and their reasons for its importance (*N* = 389).

	Response	*n* (%)
**Do you think it is important to learn about the use of antibiotics in schools?**	Yes	240 (61.7)
	No	49 (12.6)
	I don't know	100 (25.7)
	**Theme**	**Responses**, ***n*** **(%)**
**Why do you think it's important to learn about the use of antibiotics in schools**	Increasing awareness and knowledge	138 (35.5)
	Protecting health	104 (26.7)
	Appropriate use of antibiotics	55 (14.1)
	Preventing misuse	44 (11.3)
	Preventing antibiotic resistance	20 (5.1)
	Educating future generations	12 (3.1)
	Other, unclear, or no meaningful reason	146 (37.5)

Students were also invited to explain, in an open-ended question, why they considered learning about antibiotic use in schools to be important. Content analysis identified six major themes (Table 8). The most frequently reported reason was increasing awareness and knowledge (35.5%), followed by protecting individual or community health (26.7%). Other commonly reported reasons included promoting the appropriate use of antibiotics (14.1%), preventing antibiotic misuse (11.3%), preventing antibiotic resistance (5.1%), and educating future generations (3.1%). A substantial proportion of responses (37.5%) were unclear, did not provide a meaningful reason, or indicated that the participant did not know.

## 4 Discussion

This study evaluated secondary school students' knowledge and awareness of antibiotic use and antibiotic resistance (AR) in Riyadh, Saudi Arabia. While participants demonstrated positive attitudes and practices in certain areas, substantial gaps were identified in their understanding of appropriate antibiotic use and key AR concepts.

Participants demonstrated positive attitudes toward responsible antibiotic use. Most students who had recently used antibiotics obtained them through a doctor's prescription (87.4%). They also reported receiving guidance from healthcare professionals and parents, while the use of leftover antibiotics was uncommon. These findings are consistent with evidence that enforcement of prescription-only dispensing in Saudi Arabia substantially reduced inappropriate community access to antibiotics (28). However, these appropriate practices were not consistently accompanied by adequate knowledge. Fewer than half of the students understood the importance of completing the full course of antibiotic treatment, and only 30.8% correctly recognized that it is inappropriate to buy or request the same antibiotic because it had previously helped treat similar symptoms. Together, these findings suggest that prescription controls may reduce inappropriate access to antibiotics. However, they should be complemented by sustained education that support informed antibiotic-use decisions before, during, and after healthcare consultations.

Misconceptions were also evident in students' understanding of the conditions that require antibiotic treatment. The conditions most frequently considered treatable with antibiotics were cold and flu, fever, and throat infection, which are all primarily caused by viral infections. In contrast, bacterial infections such as tuberculosis, urinary tract infections (UTIs), and skin and wound infections were less frequently recognized as requiring antibiotic treatment. Similar conceptual gaps were also evident in students' understanding of antibiotic resistance. Only 19% correctly recognized that antibiotics do not treat viral infections, and just 8.7% understood that bacteria, rather than the human body, become resistant to antibiotics. Similar misconceptions have been reported among secondary school students in Saudi Arabia, Oman, Jordan, and Portugal (24, 26, 27, 29). Importantly, these misconceptions are not limited to adolescence but have also been reported among the general public, healthcare professionals, and university students in Saudi Arabia and internationally (7, 22, 30–35).

The consistency of these misconceptions across countries, age groups, and populations despite ongoing awareness initiatives suggests that existing educational campaigns and curricula may not adequately address these fundamental concepts. One possible explanation is that AR is conceptually complex, which requires understanding several interconnected concepts. These include the distinction between bacteria and viruses, the selective action of antibiotics against bacterial infections, and the evolutionary processes through which antibiotic exposure selects for resistant bacteria rather than causing the human body to become resistant (36). Therefore, educational interventions and school curricula should explicitly address these underlying microbiological and evolutionary concepts to help students develop a coherent understanding of AR (37).

Assessing students' familiarity with AR-related terminology is important as it reflects their ability to recognize and understand the language used in health communication and public awareness campaigns. Students were more familiar with terms containing the words “antibiotic” or “drug,” including “antibiotic-resistant bacteria,” “antibiotic resistance,” and “drug resistance,” than with the terms “antimicrobial resistance”, “AMR,” and “superbugs” (Figure 2). These findings are consistent with the WHO global survey, which identified antibiotic resistance, drug resistance, and antibiotic-resistant bacteria as the most familiar AR-related terms among the public (7). They are also consistent with a study among secondary school students in Oman, where antibiotic resistance and drug resistance were also the most frequently recognized terms (24). Similarly, communication research has shown that terminology containing the words antibiotic or drug is more readily understood by the public than antimicrobial resistance or its abbreviation, AMR (38, 39). Moreover, Grailey et al. (38) demonstrated that the terminology used to describe AMR also influences perceptions of the seriousness of the problem and intentions to adopt appropriate antibiotic-use behaviors, including completing prescribed antibiotic courses. These findings highlight the importance of using clear and familiar terminology when communicating AR concepts to the public and in school-based educational programs.

Doctors were the most frequently reported source of information for most AR-related terms, consistent with previous studies (7, 24, 30). In contrast, awareness campaigns were among the least frequently reported sources. Given that improving public awareness is a key objective of both the WHO Global Action Plan and the Saudi National Action Plan on AMR, these findings suggest that current awareness activities have had limited reach among secondary school students (10, 14). Future awareness initiatives should therefore consider communication strategies that better reach adolescents through settings and platforms they frequently engage with, including schools and social media, while maintaining the involvement of healthcare professionals as trusted sources of information. In addition, parents may represent an important target for future educational initiatives, as they were commonly reported as sources of guidance regarding antibiotic use.

Students generally expressed positive attitudes toward measures that help reduce AR. Most supported prescription-only antibiotic use, hand hygiene, vaccination, and shared responsibility for using antibiotics responsibly, consistent with findings among secondary school students in Oman (24). However, students showed limited awareness of the broader public health significance and personal relevance of AR. Only 27.0% recognized AR as a problem in Saudi Arabia, and fewer than one-third strongly agreed that AR is one of the world's greatest health problems. Furthermore, 68.1% believed they were not at risk of an antibiotic-resistant infection as long as they took antibiotics correctly. This finding is consistent with the limited understanding of antibiotic-resistant bacterial transmission observed in this study, where only one-third of students recognized that antibiotic-resistant bacteria can spread from person to person. Together, these findings suggest that many students did not fully appreciate that AR is a community-wide public health problem that can affect individuals regardless of their own antibiotic use. Students also expressed limited confidence in their ability to contribute to addressing AR, with 45.8% agreeing that there was little people like them could do to combat AR, while a further 38.3% neither agreed nor disagreed. Future educational interventions should therefore extend beyond improving knowledge of appropriate antibiotic use. They should also emphasize the broader public health implications of AMR, the transmission of antibiotic-resistant bacteria, and the important role of individual actions, including appropriate antibiotic use, hand hygiene, and vaccination, in limiting the spread of resistant bacteria.

In alignment with the WHO Global Action Plan, the Saudi National Action Plan recognizes antimicrobial resistance as a One Health challenge requiring coordinated action across the human, animal, agricultural, food production, and environmental sectors (14). Our findings provide insight into students' awareness of antibiotic use in food-producing animals and agriculture, an important One Health component of AR. Half of the students reported that they did not know whether antibiotics are used on a large scale in food-producing animals and agriculture in Saudi Arabia, although more than half of the students supported reducing their use in animals. Expanding AR education beyond the clinical setting to incorporate the One Health concept could help students better understand the interconnected roles of human, animal, and environmental health in the emergence and spread of AR. One Health education has been recommended as a transdisciplinary approach that should be introduced early and integrated across school subjects to strengthen understanding of complex public health challenges, including antimicrobial resistance (40). A recent study showed that integrating AMR and One Health concepts into secondary school biology education significantly improved students' engagement with and understanding of AMR (41).

Participation in science or health clubs was associated, in exploratory item-level analyses, with greater familiarity with the term antibiotic resistance, but not with any AR-concept statement after adjustment for multiple comparisons, and it was not independently associated with the composite AR-concept score after adjustment for gender and school type. Because club participation was more common in private schools (75.9% vs. 56.3%), adjustment was necessary to distinguish the contribution of extracurricular activities from that of school type. These findings provide no evidence that current science and health club activities translate into measurably better understanding of AR, even though club members are more likely to have encountered the terminology. Such clubs nevertheless represent an existing and well-attended channel through which structured AR content could be delivered to complement classroom learning, and evaluating whether targeted content delivered through them improves knowledge would be a useful next step. Students also expressed strong support for incorporating antibiotic use education into the school curriculum, with 61.7% believing this topic should be taught in schools. The qualitative findings further supported this result by demonstrating that students themselves recognized the value of school-based education. The most frequently reported reasons were to increase awareness and knowledge and to protect individual and community health. Together, these findings suggest that implementing educational initiatives would likely be well accepted by students. Studies have consistently shown that school-based AMR education improves students' knowledge, awareness, and understanding of appropriate antibiotic use and AMR (27, 29, 41). These interventions have used a variety of educational approaches, including classroom teaching, laboratory activities, interactive activities, and One Health-focused biology education. Collectively, they demonstrate the feasibility of incorporating AMR education into schools and, in some settings, have informed broader educational and national AMR initiatives (29).

Overall, our findings suggest that while Saudi Arabia's prescription-only policy has likely improved access to appropriate antibiotics, regulatory measures alone are insufficient to ensure adequate public understanding of AR and appropriate antibiotic use. The knowledge gaps identified in this study, together with students' strong support for school-based AR education, highlight the need for a coordinated educational approach. This approach should combine school curricula, healthcare professionals, families, public awareness campaigns, and science or health-related extracurricular activities to strengthen understanding of AR and promote responsible antibiotic-use behaviors from an early age.

This study has important strengths. It is among the few studies in Saudi Arabia to comprehensively assess knowledge, awareness, misconceptions, attitudes, and practices related to antibiotic use and AR among secondary school students. In addition, including students from public, private, and international schools across various regions of Riyadh provided some diversity in curriculum and setting, although the international-school group was small. However, the study also has limitations. Participating schools were recruited on a voluntary basis from a single city; students were not randomly sampled within schools, and only one international school (*n* = 11) participated. The findings should therefore be regarded as descriptive of the participating schools in Riyadh rather than representative of secondary school students in Saudi Arabia. The small international-school subgroup also meant that school-type comparisons involving this group were underpowered and imprecise. Item-level subgroup comparisons involved multiple testing and should be regarded as exploratory, and the cross-sectional design precludes causal inference regarding the association between club participation and knowledge. Students were clustered within schools, and the analyses did not account for this clustering. Finally, the questionnaire relied on self-reported responses, which might introduce recall and social desirability bias.

## Data Availability

The original contributions presented in the study are included in the article/supplementary material, further inquiries can be directed to the corresponding author.
