# Supplementary material for: Knowledge and misconceptions about antibiotic use and antibiotic resistance among secondary school students in Riyadh, Saudi Arabia: implications for school- based antibiotic resistance education
Source: Front Public Health. 2026 Sep 29;14:1962239. doi: 10.3389/fpubh.2026.1962239 (PMC13658149; doi:10.3389/fpubh.2026.1962239)
Supplement: Supplementary file 1 [file Data_Sheet_1.pdf]

Survey Questionnaire S1:

Knowledge and Misconceptions About Antibiotic Use and Resistance Among Secondary School Students in Saudi Arabia: A Baseline Assessment to Inform School-Based Antibiotic Resistance Education

عزيزي الطالب/الطالبة في المرحلة الثانوية،

أنت مدعو/ة للمشاركة في هذا الاستبيان، ونُقدّر مشاركتك كثيرًا لأنها ستساعدنا على تحقيق الهدف المنشود من هذه الدراسة.

الهدف من هذه الدراسة هو قياس مستوى المعرفة والوعي لدى طلاب وطالبات المرحلة الثانوية حول موضوع مقاومة المضادات الحيوية، وذلك من أجل تحديد المفاهيم الخاطئة والفجوات المعرفية ضمن المنهج الدراسي الحالي.

يرجى العلم بأن جميع البيانات والمعلومات التي ستقدمونها من خلال الاستبيان المرفق مع هذه الرسالة ستُعامل بسرية تامة، وستُستخدم فقط لأغراض هذه الدراسة. لن يتم جمع أي بيانات شخصية تُحدد هويتكم، وستظل هوية جميع المشاركين مجهولة.

تشاركون في هذه الدراسة بشكلٍ طوعي تمامًا، ولكم الحرية في الانسحاب منها في أي وقت دون أي تأثير سلبي. نحن نُقدّر وقتكم وجهدكم، ونؤكد لكم أن مشاركتكم ستسهم في تعزيز مجتمعنا التعليمي، وتوفير معلومات قيّمة قد تساعد في تشكيل سياسات تعليمية مستقبلية.
لأي استفسارات أو تساؤلات، يُرجى الاتصال بالباحث الرئيسي في الدراسة عبر [تفاصيل الاتصال].

Dear student,
We invite you today to participate in this survey titled "Awareness and Knowledge of Antibiotic Resistance Among High School Students." The aim of this study is to assess the level of knowledge and awareness about antimicrobial resistance among high school students in Saudi Arabia. This will help identify any misconceptions and knowledge gaps within the current science curriculum.

Please be informed that all data and information provided through the questionnaire attached to this message will be treated with the utmost confidentiality and will be used solely for the purposes of this study. No personal data that could identify you will be collected, and the identity of all participants will remain anonymous.

Your participation in this study is completely voluntary, and you are free to withdraw at any time without any negative consequences. We value your time and effort and assure you that your participation will contribute to the enhancement of our educational community and provide valuable information that may help shape future educational policies.

For any inquiries or questions, please contact the principal investigator of the study at [contact detail].
We thank you in advance for your participation and contribution.

I. THE DEMOGRAPHIC DATA OF RESPONDENTS

أولاً: البيانات الديموغرافية للمشاركين

1. What is your age?
ما هو عمرك؟

- 15
- 16
- 17
- Other: write it here---------------------

. Please write your birth month and year ---------------------
يرجى كتابة شهر وسنة ميلادك.

2. What is your gender? ما هو جنسك؟

- Male/ ذكر
- Female/ انثى

3. What is your nationality? ما هي جنسيتك؟

- Saudi/ سعودي
- Non-Saudi/ غير سعودي

4. What grade are you currently in? في أي صف دراسي أنت الآن؟

- 10/ اولى ثانوي
- 11/ ثاني ثانوي
- 12/ ثالث ثانوي

5. What type of school do you attend?
نوع المدرسة التي تدرس بها؟

- حكومية / Public
- خاصة / Private
- دولية / International

6. Do you participate in any health or science clubs at school?
هل تشارك في أي أنشطة علمية أو صحية بالمدرسة؟

- نعم / Yes
- لا / No

II. USE OF ANTIBIOTICS / استخدام المضادات الحيوية

1. When did you last take antibiotics? متى كان آخر مرة تناولت فيها المضادات الحيوية؟

- o The past month / الشهر الماضي
- o The past six months / الستة اشهر الماضية
- o The past year / العام الماضي
- o More than a year ago / منذ اكثر من عام
- o I never consumed it / لم اتناولها ابدا
- o I don't remember- I don't know / لا اتذكر - لا أعلم

2. On that occasion, did you take antibiotics from a doctor/through a medical script?
في تلك المرة، هل حصلت على المضادات الحيوية من طبيب/عبر وصفة طبية؟

○ نعم / Yes
○ لا / No
○ لا أتذكر / لا أعلم / I don't remember/I don't know

3. On that occasion, did you get advice from a doctor, nurse or pharmacist on how to take them?
في تلك المرة، هل تلقيت نصيحة من طبيب أو ممرضة أو صيدلي حول كيفية تناولها؟

○ نعم، تلقيت تعليمات (على سبيل المثال: مع الطعام، لمدة سبعة أيام) / Yes, I got instructions (for example: with food, for seven days)
○ لا / No
○ لا أتذكر / لا أعلم / I don't remember/I don't know

4. On that occasion, did you get instructions from your parents about how to consume these antibiotics?
في تلك المرة، هل تلقيت تعليمات من والديك حول كيفية تناول هذه المضادات الحيوية؟

○ نعم، تلقيت تعليمات (على سبيل المثال: مع الطعام، لمدة سبعة أيام) / Yes, I got instructions (for example: with food, for seven days)
○ لا / No
○ لا أتذكر / لا أعلم / I don't remember/I don't know

5. On that occasion, where did you/your parents obtain these antibiotics from?
في تلك المرة، من أين حصلت أنت/والديك على هذه المضادات الحيوية؟

○ لا أتذكر / Can't remember
○ من مكان/شخص آخر / Somewhere/someone else
○ كانت مخزنة لدي من مرة سابقة / I had them saved up from a previous time

o من صديق أو فرد من العائلة / Friend or family member
o الإنترنت / The internet
o من متجر طبي أو صيدلية / Medical store or pharmacy

6. When do you think you should stop taking antibiotics once you've begun treatment? متى تعتقد أنه يجب عليك التوقف عن تناول المضادات الحيوية بعد بدء العلاج؟

- o When I feel better / عندما أشعر بتحسن
- o When I have antibiotics according to instructions / عندما أتناول المضادات الحيوية وفقًا للتعليمات
- o I don't know / لا أعلم

III. KNOWLEDGE OF ANTIBIOTICS & ANTIBIOTIC RESISTANCE

الوعي بمقاومة المضادات الحيوية

7. It's okay to use antibiotics that were given to a friend or family member, as long as they were used to treat the same illness

لا مانع من استخدام المضادات الحيوية التي أُعطيت لصديق أو فرد من العائلة، طالما أنها استخدمت لعلاج نفس المرض

- o Don't know / لا أعلم
- o TRUE / صحيح
- o FALSE / خطأ

8. It's okay to buy the same antibiotics, or request these from a doctor, if you're sick and they helped you get better when you had the same symptoms before

لا مانع من شراء نفس المضادات الحيوية، أو طلبها من الطبيب، إذا كنت مريضًا وساعدتك على الشفاء عندما كانت لديك نفس الأعراض من قبل

- o Don't know / لا أعلم
- o TRUE / صحيح
- o FALSE / خطأ

9. Which of the following diseases can be cured with antibiotics? Choose all the answers that you think are correct.

أي من الأمراض التالية يمكن علاجها بالمضادات الحيوية؟ اختر جميع الإجابات التي تعتقد أنها صحيحة.

- o Acquired Immune Deficiency Syndrome (AIDS) / متلازمة نقص المناعة المكتسبة (الإيدز) (وهي عدوى فيروسية تصيب جهاز المناعة بالجسم، مما يجعل من الصعب مكافحة الأمراض)

- o Gonorrhoea (Infection that's passed between people through sexual contact. It's caused by bacteria and can lead to pain and other health problems if not treated) / السيلان (عدوى بكتيرية تنتقل بالاتصال الجنسي)

- Urinary tract infection / التهاب المسالك البولية
- Diarrhoea / الإسهال
- Cold & flu / نزلات البرد والإنفلونزا
- Fever / الحمى
- Malaria (life-threatening disease caused by parasites and spread by some types of mosquitoes) / (مرض مهدد للحياة تسببه الطفيليات وينتقل عن طريق بعض أنواع البعوض)الملاريا
- Measles / الحصبة
- Skin infection/wound infection / التهاب الجلد/الجروح
- Throat infection / التهاب الحلق
- Body aches / آلام الجسم
- Headache / الصداع
- Tuberculosis / السل
- Brucellosis: bacterial infection caused by direct contact with infected animals or by eating or drinking contaminated animal products. / داء البروسيلا: عدوى بكتيرية تنتقل من الحيوانات إلى الإنسان عن طريق تناول مشتقات الحليب غير المبسترة أو من خلال ملامسة الحيوانات المصابة.

10. "Have you ever heard of the term "Antibiotic resistance?"

هل سمعت بمصطلح "مقاومة المضادات الحيوية" من قبل؟

- Yes / نعم
- No / لا

If you answered yes, from where did you hear about this term? Choose all possible answers.

من أين سمعت بهذا المصطلح؟ اختر جميع الإجابات الممكنة

- Doctor / طبيب
- Nurse / ممرض/ممرضة
- Pharmacist / صيدلي
- School curriculum / المنهج الدراسي
- Friend or relative / صديق أو قريب
- Media (TV, radio, newspapers or other, including social media) / وسائل الإعلام (التلفزيون، الراديو، الصحف أو غيرها، بالإضافة إلى وسائل التواصل الاجتماعي)

- Awareness campaign / حملة توعية
- I don't remember / لا أتذكر
- Other / أخرى: ______________________

11. "Have you ever heard about the term "Super Bugs" ?

هل سمعت من قبل بمصطلح "البكتيريا الخارقة"؟

- Yes / نعم
- No / لا

. If you answered yes, from where did you hear about this term? Choose all possible answers.

من أين سمعت بهذا المصطلح؟ اختر جميع الإجابات الممكنة

- Doctor / طبيب
- Nurse / ممرض/ممرضة
- Pharmacist / صيدلي
- School curriculum / المنهج الدراسي
- Friend or relative / صديق أو قريب
- Media (TV, radio, newspapers or other, including social media) / وسائل الإعلام (التلفزيون، الراديو، الصحف أو غيرها، بالإضافة إلى وسائل التواصل الاجتماعي)
- Awareness campaign / حملة توعية
- I don't remember / لا أتذكر
- Other / أخرى: ______________________

12. "Have you ever heard of the term "Drug resistance?"

هل سمعت بمصطلح "مقاومة الأدوية" من قبل؟

- Yes / نعم
- No / لا

If you answered yes, from where did you hear about this term? Choose all possible answers.

من أين سمعت بهذا المصطلح؟ اختر جميع الإجابات الممكنة

- Doctor / طبيب
- Nurse / ممرض/ممرضة
- Pharmacist / صيدلي
- School curriculum / المنهج الدراسي
- Friend or relative / صديق أو قريب

- Media (TV, radio, newspapers or other, including social media) / وسائل الإعلام (التلفزيون، الراديو، الصحف أو غيرها، بالإضافة إلى وسائل التواصل الاجتماعي)
- Awareness campaign / حملة توعية
- I don't remember / لا أتذكر
- Other / أخرى: ______________________

13. Have you ever heard about the term "antimicrobial resistance?"

هل سمعت من قبل بمصطلح "مقاومة مضادات الميكروبات"؟

- Yes / نعم
- No / لا

If you answered yes, from where did you hear about this term? Choose all possible answers.

من أين سمعت بهذا المصطلح؟ اختر جميع الإجابات الممكنة

- Doctor / طبيب
- Nurse / ممرض/ممرضة
- Pharmacist / صيدلي
- School curriculum / المنهج الدراسي
- Friend or relative / صديق أو قريب
- Media (TV, radio, newspapers or other, including social media) / وسائل الإعلام (التلفزيون، الراديو، الصحف أو غيرها، بالإضافة إلى وسائل التواصل الاجتماعي)
- Awareness campaign / حملة توعية
- I don't remember / لا أتذكر
- Other / أخرى: ______________________

14. "Have you ever heard about the term "AMR"?

هل سمعت من قبل بمصطلح "AMR"؟

- Yes / نعم
- No / لا

If you answered yes, from where did you hear about this term? Choose all possible answers.

من أين سمعت بهذا المصطلح؟ اختر جميع الإجابات الممكنة

- Doctor / طبيب
- Nurse / ممرض/ممرضة
- Pharmacist / صيدلي
- School curriculum / المنهج الدراسي

- Friend or relative / صديق أو قريب
- Media (TV, radio, newspapers or other, including social media) / وسائل الإعلام (التلفزيون، الراديو، الصحف أو غيرها، بالإضافة إلى وسائل التواصل الاجتماعي)
- Awareness campaign / حملة توعية
- I don't remember / لا أتذكر
- Other / أخرى: ______________________

15. "Have you ever heard about the term "antibiotic-resistant bacteria"?

هل سمعت من قبل بمصطلح "البكتيريا المقاومة للمضادات الحيوية"؟

- Yes / نعم
- No / لا

If you answered yes, from where did you hear about this term? Choose all possible answers.

من أين سمعت بهذا المصطلح؟ اختر جميع الإجابات الممكنة

- Doctor / طبيب
- Nurse / ممرض/ممرضة
- Pharmacist / صيدلي
- School curriculum / المنهج الدراسي
- Friend or relative / صديق أو قريب
- Media (TV, radio, newspapers or other, including social media) / وسائل الإعلام (التلفزيون، الراديو، الصحف أو غيرها، بالإضافة إلى وسائل التواصل الاجتماعي)
- Awareness campaign / حملة توعية
- I don't remember / لا أتذكر
- Other / أخرى: ______________________

16. Please choose either "Correct", "Wrong" or "I Don't know" to indicate your answer to the following expressions.

يرجى اختيار "صح" أو "خطأ" أو "لا أعلم" للدلالة على إجابتك للعبارات التالية.

No.	Statement / العبارة	Correct / صح	Wrong / خطأ	I Don't know / لا أعلم
a	Antibiotic resistance occurs when your body becomes resistant to antibiotics and they no longer work as well مقاومة المضادات الحيوية تحدث عندما يصبح الجسم مقاومًا للمضادات الحيوية وبالتالي لا تعمل هذه المضادات بشكل جيد	☐	☐	☐
b	A lot of types of infections have become increasingly resistant to antibioticsالعديد من أنواع العدوى الميكروبية أصبحت أكثر مقاومة للمضادات الحيوية	☐	☐	☐
c	If bacteria are resistant to antibiotics, it can be very difficult or impossible to treat the infections they cause إذا كانت البكتيريا مقاومة للمضادات الحيوية، فقد يكون من الصعب جدًا أو مستحيلًا علاج العدوى التي تسببها.	☐	☐	☐
d	Antibiotics resistance is an issue that could affect me or my family مقاومة المضادات الحيوية هي مشكلة قد تؤثر علي أو على عائلتي.	☐	☐	☐
e	Antibiotics resistance is an issue in other countries but not in Saudi Arabiaمقاومة المضادات الحيوية هي مشكلة في دول أخرى ولكن ليس في السعودية	☐	☐	☐
f	Antibiotic resistance is only a problem for people who take antibiotics regularly مقاومة المضادات الحيوية هي مشكلة فقط للأشخاص الذين يتناولون المضادات الحيوية بشكل متكرر	☐	☐	☐
g	Bacteria which are resistant to antibiotics can be spread from person to personالبكتيريا المقاومة للمضادات الحيوية يمكن أن تنتقل من شخص لآخر	☐	☐	☐
h	Antibiotic-resistant infections could make medical procedures like surgery, organ transplants and cancer treatment much more dangerous العدوى المقاومة للمضادات الحيوية يمكن أن تجعل الإجراءات الطبية مثل الجراحة وزراعة الأعضاء وعلاج السرطان أكثر خطورة	☐	☐	☐
i	Antibiotic resistance can be overtaken by increasing the dose of the medicationيمكن التغلب على مقاومة المضادات الحيوية بزيادة جرعة الدواء	☐	☐	☐
j	It is possible to cure diseases related to viruses through using antibiotics من الممكن علاج الأمراض المتعلقة بالفيروسات باستخدام المضادات الحيوية	☐	☐	☐

17. **To what extent do you agree with the following statements related to how to address the issue of antibiotic resistance?**

ما مدى موافقتك على المعلومات التالية المتعلقة بكيفية معالجة مشكلة مقاومة المضادات الحيوية؟

**no.**	**Statement / العبارة**	**Agree strongly موافق بشدة**	**Agree slightly موافق بعض الشيء**	**Neither agree nor disagree لا أوافق ولا أعارض**	**Disagree slightly غير موافق بعض الشيء**	**Disagree strongly غير موافق بشدة**
a	People have to use antibiotics when they are prescribed by a doctor only يجب على الناس استخدام المضادات الحيوية عندما يصفها الطبيب فقط	☐	☐	☐	☐	☐
**b**	Farmers should give fewer antibiotics to food-producing animals يجب على المزارعين إعطاء كميات أقل من المضادات الحيوية للحيوانات المنتجة للغذاء	☐	☐	☐	☐	☐
**c**	People must not keep leftover antibiotics and must not use it on other occasions to treat other illnesses يجب على الناس عدم الاحتفاظ بالمضادات الحيوية المتبقية وعدم استخدامها في مناسبات أخرى لعلاج أمراض أخرى	☐	☐	☐	☐	☐
**d**	Parents should make sure all of their children's vaccinations are up-to-date يجب على الوالدين التأكد من تحديث جميع تطعيمات أطفالهم	☐	☐	☐	☐	☐
**e**	People should wash their hands regularly يجب على الناس غسل أيديهم بانتظام	☐	☐	☐	☐	☐
**f**	Doctors must prescribe antibiotics only when necessary يجب على الأطباء وصف المضادات الحيوية فقط عند الضرورة	☐	☐	☐	☐	☐
**g**	The government must provide rewards for those who develop new antibiotics يجب على الحكومة تقديم مكافآت لأولئك الذين يطورون مضادات حيوية جديدة	☐	☐	☐	☐	☐
**h**	Pharmaceutical companies must develop new antibiotics يجب على الشركات الصيدلانية تطوير مضادات حيوية جديدة	☐	☐	☐	☐	☐

18. To what extent do you agree with the following statements?

ما مدى موافقتك على المعلومات التالية؟

No.	Statement / العبارة	Agree strongly موافق بشدة	Agree slightly موافق بعض الشيء	Neither agree nor disagree لا أوافق ولا أعارض	Disagree slightly غير موافق بعض الشيء	Disagree strongly غير موافق بشدة
a	Antibiotic resistance is one of the biggest problems facing the world مقاومة المضادات الحيوية هي واحدة من أكبر المشاكل التي تواجه العالم	☐	☐	☐	☐	☐
b	Medical experts are able to find a solution to antibiotic resistance before it becomes too serious الخبراء الطبيون قادرون على إيجاد حل لمقاومة المضادات الحيوية قبل أن تصبح خطيرة جداً.	☐	☐	☐	☐	☐
c	Everyone needs to take responsibility for using antibiotics responsibly الجميع بحاجة إلى تحمّل المسؤولية عند استخدام المضادات الحيوية واستعمالها بشكل مسؤول.	☐	☐	☐	☐	☐
d	There isn't much that people like me can do to stop the problem of antibiotic resistance ليس هناك الكثير الذي يمكن لأشخاص مثلي فعله لوقف مشكلة مقاومة المضادات الحيوية.	☐	☐	☐	☐	☐
e	I am worried about the impact that antibiotic resistance will have on my health, and that of my family أنا قلق بشأن التأثير الذي ستحدثه مقاومة المضادات الحيوية على صحتي وصحة عائلتي.	☐	☐	☐	☐	☐
f	I am not at risk of getting an antibiotic-resistant infection, as long as I take my antibiotics correctly أنا لست في خطر الإصابة بعدوى مقاومة للمضادات الحيوية، طالما أنني أتناول المضادات الحيوية بشكل صحيح	☐	☐	☐	☐	☐

19. Do you think that antibiotics are used in a large scale in the agricultural sector (including the sector of food-producing animals) in Saudi Arabia?
هل تعتقد أن المضادات الحيوية تُستخدم على نطاق واسع في القطاع الزراعي (بما في ذلك قطاع الحيوانات المنتجة للغذاء) في المملكة العربية السعودية؟

- o نعم / Yes
- o لا / No
- o لا أعلم / I don't know

20. Do you think it is important to learn about the use of antibiotics in schools?
هل تعتقد أنه من المهم تعلم استخدام المضادات الحيوية في المدارس؟

- نعم / Yes
- لا / No
- لا أعلم / I don't know

---

21. Why do you think it's important to learn about the use of antibiotics in schools?
لماذا تعتقد أنه من المهم تعلم استخدام المضادات الحيوية في المدارس؟

………
………
………
………
…………
