# Supplementary material for: Knowledge and misconceptions about antibiotic use and antibiotic resistance among secondary school students in Riyadh, Saudi Arabia: implications for school- based antibiotic resistance education
Source: Front Public Health. 2026 Sep 29;14:1962239. doi: 10.3389/fpubh.2026.1962239 (PMC13658149; doi:10.3389/fpubh.2026.1962239)
Supplement: Supplementary file 2 [file Data_Sheet_2.docx]

Supplementary Material

**Supplementary Figure S1**. Sources of information for antibiotic resistance-related terms among students who were aware of each term. Percentages represent the proportion of students who reported each source of information and were calculated using the number of students familiar with each term as the denominator. Participants could select more than one source of information. Media includes television, radio, newspapers, and social media.

**Supplementary Tables**

| **Table S1**. Familiarity with antibiotic resistance-related terminology according to participant characteristics (N = 389) | | | | | | | | | | | | | | |
| --- | --- | --- | --- | --- | --- | --- | --- | --- | --- | --- | --- | --- | --- | --- |
| **Term** | **Overall**  **n (%)** | **Female (n = 191)** | **Male (n = 198)** | **p** | **q** | **International (n = 11)** | **Private (n = 108)** | **Public (n = 270)** | **p** | **q** | **Club: No (n = 151)** | **Club: Yes (n = 238)** | **p** | **q** |
| Antibiotic resistance | 173 (44.5) | 77 (40.3) | 96 (48.5) | 0.126 | 0.373 | 5 (45.5) | 44 (40.7) | 124 (45.9) | 0.656 | 0.819 | 53 (35.1) | 120 (50.4) | 0.003 | 0.035 |
| Superbugs | 92 (23.7) | 42 (22.0) | 50 (25.3) | 0.475 | 0.783 | 3 (27.3) | 18 (16.7) | 71 (26.3) | 0.132 | 0.373 | 29 (19.2) | 63 (26.5) | 0.112 | 0.373 |
| Drug resistance | 158 (40.6) | 74 (38.7) | 84 (42.4) | 0.472 | 0.783 | 7 (63.6) | 42 (38.9) | 109 (40.4) | 0.278 | 0.641 | 50 (33.1) | 108 (45.4) | 0.020 | 0.188 |
| Antimicrobial resistance | 84 (21.6) | 37 (19.4) | 47 (23.7) | 0.325 | 0.658 | 2 (18.2) | 22 (20.4) | 60 (22.2) | 0.890 | 0.977 | 26 (17.2) | 58 (24.4) | 0.102 | 0.369 |
| AMR | 85 (21.9) | 38 (19.9) | 47 (23.7) | 0.391 | 0.735 | 2 (18.2) | 23 (21.3) | 60 (22.2) | 0.938 | 0.980 | 26 (17.2) | 59 (24.8) | 0.101 | 0.369 |
| Antibiotic-resistant bacteria | 192 (49.4) | 94 (49.2) | 98 (49.5) | 1.000 | 1.000 | 5 (45.5) | 46 (42.6) | 141 (52.2) | 0.231 | 0.571 | 66 (43.7) | 126 (52.9) | 0.078 | 0.346 |
| p-values are from Fisher's exact test. q-values are Benjamini–Hochberg adjusted p-values across all 47 subgroup comparisons in Tables 3, S1 and S2. The international-school subgroup comprised 11 students; percentages for this group are imprecise and school-type p-values should be interpreted with caution. | | | | | | | | | | | | | | |

| **Table S2.** Association between participant characteristics and correct responses to antibiotic resistance knowledge statements | | | | | | |
| --- | --- | --- | --- | --- | --- | --- |
| **Statement** | **Correct response** | **Characteristic** | **Category (N)** | **n (%) correct** | **p** | **q** |
| Antibiotic resistance occurs when your body becomes resistant  to antibiotics and they no longer work as well. | Wrong | School type | International (11) | 0 (0.0) | 0.508 | 0.796 |
|  |  |  | Private (108) | 11 (10.2) |  |  |
|  |  |  | Public (270) | 23 (8.5) |  |  |
|  |  |  |  |  |  |  |
|  |  | Science/Health club participation | No (151) | 8 (5.3) | 0.066 | 0.346 |
|  |  |  | Yes (238) | 26 (10.9) |  |  |
| A lot of types of infections have become increasingly resistant  to antibiotics. | Correct | School type | International (11) | 5 (45.5) | 0.884 | 0.977 |
|  |  |  | Private (108) | 41 (38.0) |  |  |
|  |  |  | Public (270) | 103 (38.1) |  |  |
|  |  |  |  |  |  |  |
|  |  | Science/Health club participation | No (151) | 47 (31.1) | 0.025 | 0.188 |
|  |  |  | Yes (238) | 102 (42.9) |  |  |
| If bacteria are resistant to antibiotics, it can be very difficult or impossible  to treat the infections they cause. | Correct | School type | International (11) | 7 (63.6) | 0.336 | 0.658 |
|  |  |  | Private (108) | 44 (40.7) |  |  |
|  |  |  | Public (270) | 113 (41.9) |  |  |
|  |  |  |  |  |  |  |
|  |  | Science/Health club participation | No (151) | 61 (40.4) | 0.599 | 0.819 |
|  |  |  | Yes (238) | 103 (43.3) |  |  |
| Antibiotic resistance is an issue that could affect me or my family. | Correct | School type | International (11) | 4 (36.4) | 0.613 | 0.819 |
|  |  |  | Private (108) | 47 (43.5) |  |  |
|  |  |  | Public (270) | 103 (38.1) |  |  |
|  |  |  |  |  |  |  |
|  |  | Science/Health club participation | No (151) | 58 (38.4) | 0.750 | 0.882 |
|  |  |  | Yes (238) | 96 (40.3) |  |  |
| Antibiotic resistance is an issue in other countries but not in Saudi Arabia. | Wrong | School type | International (11) | 5 (45.5) | 0.143 | 0.373 |
|  |  |  | Private (108) | 34 (31.5) |  |  |
|  |  |  | Public (270) | 66 (24.4) |  |  |
|  |  |  |  |  |  |  |
|  |  | Science/Health club participation | No (151) | 44 (29.1) | 0.483 | 0.783 |
|  |  |  | Yes (238) | 61 (25.6) |  |  |
| Antibiotic resistance is only a problem for people who take antibiotics regularly. | Wrong | School type | International (11) | 0 (0.0) | 0.290 | 0.641 |
|  |  |  | Private (108) | 19 (17.6) |  |  |
|  |  |  | Public (270) | 40 (14.8) |  |  |
|  |  |  |  |  |  |  |
|  |  | Science/Health club participation | No (151) | 23 (15.2) | 1.00 | 1.000 |
|  |  |  | Yes (238) | 36 (15.1) |  |  |
| Bacteria that are resistant to antibiotics can be spread from person to person. | Correct | School type | International (11) | 3 (27.3) | 0.894 | 0.977 |
|  |  |  | Private (108) | 37 (34.3) |  |  |
|  |  |  | Public (270) | 92 (34.1) |  |  |
|  |  |  |  |  |  |  |
|  |  | Science/Health club participation | No (151) | 41 (27.2) | 0.028 | 0.188 |
|  |  |  | Yes (238) | 91 (38.2) |  |  |
| Antibiotic-resistant infections could make medical procedures such as surgery, organ transplantation, and cancer treatment much more dangerous. | Correct | School type | International (11) | 6 (54.5) | 0.568 | 0.819 |
|  |  |  | Private (108) | 45 (41.7) |  |  |
|  |  |  | Public (270) | 106 (39.3) |  |  |
|  |  |  |  |  |  |  |
|  |  | Science/Health club participation | No (151) | 59 (39.1) | 0.751 | 0.882 |
|  |  |  | Yes (238) | 98 (41.2) |  |  |
| Antibiotic resistance can be overcome by increasing the dose of the medication. | Wrong | School type | International (11) | 2 (18.2) | 0.081 | 0.346 |
|  |  |  | Private (108) | 46 (42.6) |  |  |
|  |  |  | Public (270) | 87 (32.2) |  |  |
|  |  |  |  |  |  |  |
|  |  | Science/Health club participation | No (151) | 50 (33.1) | 0.662 | 0.819 |
|  |  |  | Yes (238) | 85 (35.7) |  |  |
| Viral infections can be cured using antibiotics. | Wrong | School type | International (11) | 2 (18.2) | 0.915 | 0.977 |
|  |  |  | Private (108) | 22 (20.4) |  |  |
|  |  |  | Public (270) | 50 (18.5) |  |  |
|  |  |  |  |  |  |  |
|  |  | Science/Health club participation | No (151) | 31 (20.5) | 0.597 | 0.819 |
|  |  |  | Yes (238) | 43 (18.1) |  |  |
| Values are presented as n (%). Percentages were calculated using the number of students within each subgroup as the denominator. Statements marked “Wrong” are misconceptions; the correct response is to reject them. Percentages represent correct responses in either direction. “I don’t know” responses were scored as incorrect. p-values are from Fisher's exact test; q-values are Benjamini–Hochberg adjusted p-values across all 47 subgroup comparisons in Tables 3, S1 and S2. The international-school subgroup comprised 11 students; percentages for this group are imprecise, and school-type p-values should be interpreted with caution | | | | | | |

.

| **Table S3**: Students' Attitudes toward how to address the issue of antibiotic resistance (N=389) | | | | | | |
| --- | --- | --- | --- | --- | --- | --- |
| **Statement** | **Agree (Strongly + Slightly)**  **n (%)** | **Agree strongly**  **n (%)** | **Agree slightly n (%)** | **Neither agree nor disagree**  **n (%)** | **Disagree slightly**  **n (%)** | **Disagree strongly**  **n (%)** |
| People should use antibiotics only when prescribed by a doctor. | 354 (91.0) | 261 (67.1) | 93 (23.9) | 23 (5.9) | 3 (0.8) | 9 (2.3) |
| Farmers should give fewer antibiotics to food-producing animals. | 232 (59.6) | 135 (34.7) | 97 (24.9) | 131 (33.7) | 16 (4.1) | 10 (2.6) |
| People should not keep leftover antibiotics or use them to treat other illnesses. | 261 (67.1) | 195 (50.1) | 66 (17.0) | 73 (18.8) | 31 (8.0) | 24 (6.2) |
| Parents should ensure that all their children's vaccinations are up to date. | 345 (88.7) | 310 (79.7) | 35 (9.0) | 36 (9.3) | 2 (0.5) | 6 (1.5) |
| People should wash their hands regularly. | 352 (90.5) | 320 (82.3) | 32 (8.2) | 26 (6.7) | 6 (1.5) | 5 (1.3) |
| Doctors should prescribe antibiotics only when necessary. | 294 (75.6) | 204 (52.4) | 90 (23.1) | 65 (16.7) | 19 (4.9) | 11 (2.8) |
| The government should provide rewards for developing new antibiotics. | 311 (79.9) | 234 (60.2) | 77 (19.8) | 65 (16.7) | 6 (1.5) | 7 (1.8) |
| Pharmaceutical companies should develop new antibiotics. | 307 (78.9) | 216 (55.5) | 91 (23.4) | 61 (15.7) | 12 (3.1) | 9 (2.3) |

| **Table S4:** Students' attitudes toward the importance of antibiotic resistance | | | | | | |
| --- | --- | --- | --- | --- | --- | --- |
| **Statement** | **Agree (Strongly + Slightly) n (%)** | **Agree**  **strongly n (%)** | **Agree**  **slightly n (%)** | **Neither agree**  **nor disagree n (%)** | **Disagree**  **slightly n (%)** | **Disagree**  **strongly n (%)** |
| Antibiotic resistance is one of the biggest problems facing the world. | 227 (58.4) | 115 (29.6) | 112 (28.8) | 134 (34.4) | 19 (4.9) | 9 (2.3) |
| Medical experts will find a solution to antibiotic resistance before it becomes too serious. | 282 (72.5) | 158 (40.6) | 124 (31.9) | 82 (21.1) | 15 (3.9) | 10 (2.6) |
| Everyone should take responsibility for using antibiotics responsibly. | 308 (79.2) | 235 (60.4) | 73 (18.8) | 61 (15.7) | 14 (3.6) | 6 (1.5) |
| There is little that people like me can do to stop antibiotic resistance. | 178 (45.8) | 107 (27.5) | 71 (18.3) | 149 (38.3) | 45 (11.6) | 17 (4.4) |
| I am worried about the impact of antibiotic resistance on my health and my family's health. | 264 (67.9) | 167 (42.9) | 97 (24.9) | 99 (25.4) | 15 (3.9) | 11 (2.8) |
| I am not at risk of getting an antibiotic-resistant infection as long as I take my antibiotics correctly. | 265 (68.1) | 172 (44.2) | 93 (23.9) | 89 (22.9) | 23 (5.9) | 12 (3.1) |
